# Prevalence and validity of self-reported smoking in Indigenous and non-Indigenous young adults in the Australian Northern Territory

**DOI:** 10.1186/1471-2458-14-861

**Published:** 2014-08-21

**Authors:** Mark S Pearce, Kay D Mann, Gurmeet Singh, Belinda Davison, Susan M Sayers

**Affiliations:** Institute of Health & Society, Newcastle University, Sir James Spence Institute, Royal Victoria Infirmary, Newcastle upon Tyne, UK; Menzies School of Health Research, Charles Darwin University, Darwin, Australia

**Keywords:** Cigarette smoking, Validation, Cotinine, Aboriginal Australians, Passive smoking

## Abstract

**Background:**

In this study, we used data from Australia’s Northern Territory to assess differences in self-reported smoking prevalence between the Indigenous and non-Indigenous populations. We also used urinary cotinine data to assess the validity of using self-reported smoking data in these populations.

**Methods:**

The Aboriginal Birth Cohort (ABC) is a prospective study of 686 Aboriginal babies born in Darwin 1987–90. The Top End Cohort (TEC) is a study of non-Indigenous adolescents, all born in Darwin 1987–91. In both studies, participants aged between 16 and 21 years, were asked whether they smoked. Urinary cotinine measurements were made from samples taken at the same visits.

**Results:**

Self-reported smoking prevalence was 68% in the ABC and 14% in the TEC. Among the self-reported non-smokers, the median cotinine levels were higher in the ABC (33 ng/ml) than in the TEC (5 ng/ml), with greater percentages of reported non-smokers in the under 50 ng/ml group in the TEC than in the ABC

**Conclusions:**

Prevalence of smoking was much higher in the ABC than in the TEC. The higher cotinine levels in ABC non-smokers may reflect an underestimated prevalence, but is also likely to reflect higher levels of passive smoking. A broader approach encompassing social, cultural and language factors with increased attention to smoking socialisation factors is required.

## Background

Tobacco smoking is the single most preventable cause of ill health and death in Australia and is estimated to be the cause of 15,000 deaths in the country per year [1]. Although smoking rates are declining currently in both Indigenous and non-Indigenous people in Australia, marked discrepancies remain between Indigenous and non-Indigenous rates and their rates of decline [2]. After adjusting for differences in the age structures of the two populations, daily smoking was 2.2 times more common among Indigenous people aged 15 years or older in 2010 than among their non-Indigenous counterparts (38% and 18%, respectively [3]). Furthermore, a comparison of trends of smoking prevalence among Indigenous and non-Indigenous Australian secondary school students showed that rates declined between 1996 and 2005 for both groups [4]. However, the rate of decline differed, with a steady decline across the entire study period for the non-Indigenous students compared to a decline only between 1999 and 2002 for Indigenous students [4]. The majority of cigarette smokers begin smoking in childhood or adolescence [5], with smoking initiation usually completed by 25 years of age [6, 7]. In order to inform different intervention strategies to further reduce initiation and prevalence of smoking in young Australians, it is important to explore the differences in smoking prevalence between adolescent non-Indigenous and Indigenous Australians.

Smoking behaviours are most often assessed by questionnaire, which is prone to inaccurate responses and underestimation of the true prevalence of cigarette smoking [8–10]. This has prompted a number of studies to attempt to validate smoking questionnaire responses, for example by using exhaled carbon monoxide [10–12], serum, plasma or urine levels of nicotine [13], cotinine [13–19] or thiocyanate [11], the bogus pipeline approach [20], or store turnover of cigarettes, as was done previously in the context of remote Indigenous Australians [21]. Among the findings from such studies have been differences in the validity of self-reported smoking by sex [10, 19] and ethnicity [19]. Therefore, in addition to assessing whether smoking prevalence differs between different population groups, it is also crucial to assess whether differences in reliability also exist in order to assess the validity of the prevalence estimates.

In this study we used data and samples from two cohorts, the Indigenous Aboriginal Birth Cohort (ABC) [22, 23] and the non-Indigenous Top End Cohort (TEC) [24] with two aims; i) to assess smoking prevalence from self-report questionnaire data and, ii) to assess validity of self-reported smoking, using urinary measures of cotinine. Both cohorts are based in the Australian Northern Territory (NT).

## Methods

The Life Course Program based at the Menzies School of Health Research is composed of two distinct, but complementary, cohorts. The ABC is a prospective study of 686 Aboriginal babies (a representative samples of the 1238 eligible babies) recruited at Royal Darwin Hospital between January 1987 and March 1990 to a mother recorded as Aboriginal in the Delivery Suite Register [22]. Data used in this analysis were obtained during follow-up between December 2005 and January 2008, in over 40 different locations in the ‘Top End’ of the NT [23].

The TEC is a study of 195 non-Indigenous adolescents, age- and sex-matched to the ABC study participants, born in Darwin between 1987 and 1991 (contemporaneous interval with the ABC) and still residing in non-remote locations within the Top End at the time of recruitment [24]. The numbers of non-Indigenous people in remote areas of the NT are very few and this is largely a transient population. Recruitment occurred between November 2007 and September 2009 and involved young people from secondary and tertiary schools, as well as those in employment and unemployed. Inclusion was limited to people born to non-Indigenous mothers as the ABC study encompassed those people. Both studies were approved by the Human Research Ethics Committee (HREC) of the Northern Territory Department of Health and the Menzies School of Health Research (ABC reference number 05/26, TEC reference number 07/20). The ABC study also obtained approval from the Aboriginal Ethical Sub-committee which has the power of veto.

The residence of ABC participants was classified as ‘non-remote’ (living within the urban areas of Darwin and its satellite city Palmerston) and ‘remote’ (living in a rural community with an Aboriginal council, a rural town, or a town camp). All TEC participants resided in urban areas of Darwin and its satellite city Palmerston, hence were all ‘non-remote’.

Both the ABC and TEC underwent a comprehensive health check with cohort members aged between 16 and 21 years.

### Self-reported smoking

For both the ABC and TEC, details on smoking status (current smoker or non-smoker) were self-reported as part of a lifestyle questionnaire (Appendix) including smoking of tobacco and marijuana and consumption of alcohol. The questions used were chosen as they had been developed and used previously in a remote Aboriginal setting [25]. The participants had the choice of answering the questionnaires on a laptop or a paper version. Participants, particularly those in remote areas, answered these questions as they were read out to them by the study team.

### Measurement of cotinine

During the medical examinations, questionnaires were completed, urine samples were collected and cotinine levels were measured. A cut-off of 50 ng/ml was used to verify non-smokers in accordance with previously published papers [14, 17, 19]. Further cut-off points of 170, 550 and 2100 ng/ml, as used by Zielińska-Danch et al. [17], have been used for descriptive purposes.

### Statistical analysis

Prevalence/percentage figures with 95% confidence intervals (95% CI) were calculated and chi-squared tests for associations between smoking and cohort, sex and residential region (ABC only) were done using a p-value less than 0.05 for statistical significance. Urinary cotinine was treated as a continuous measurement and all other variables were treated as categorical. Comparisons of cotinine levels between groups were done using the Wilcoxon rank-sum test. All statistical analyses were done using the Stata statistical software package, version 12.0 (StataCorp, College Station, TX).

## Results

Self-reported smoking data were available for 407 (197 male) participants of the ABC and for 182 (78 male) of the TEC. Average age at assessment for those with smoking data was similar between the cohorts (mean (sd) was 17.9 (1.13) for the ABC and 18.1 (1.38) for the TEC). Self-reported smoking prevalence was significantly different between the cohorts (p < 0.0001), with 68% prevalence in the ABC and 14% prevalence in the TEC (Table 1). Prevalence rates were similar for males and females (*χ*^2^ = 0.26, df = 1, p = 0.61 for the ABC and *χ*^2^ = 0.10, df = 1, p = 0.76 for the TEC) and also for remote and non-remote residential areas in the ABC (*χ*^2^ = 1.06, df = 1, p = 0.30). Six participants in the ABC (1.5%) and 12 in the TEC used to smoke, but were current non-smokers. No-one in either cohort reported using nicotine replacement therapy at the time of the study.

**Table 1:** **Self-reported smoking prevalence by cohort, sex and, for the ABC only, residential area**

	Self-reported smoking		
	n	Prevalence	95% CI
**ABC**			
Overall	407	0.68	0.63, 0.72
Men	197	0.69	0.63, 0.76
Women	210	0.67	0.60, 0.73
Remote residence	320	0.69	0.64, 0.75
Non-remote residence	85	0.64	0.54, 0.74
**TEC**			
Overall	182	0.14	0.08, 0.19
Men	78	0.13	0.03, 0.21
Women	104	0.14	0.06, 0.22

95% CI: 95% Confidence Interval.

Valid urinary cotinine measurements were available for 391 of the ABC and 156 of the TEC. Descriptive summaries of the median cotinine levels by self-reported smoking status are given in Table 2, by cohort, sex and, for the ABC only, residential area. Among the self-reported smokers, the median cotinine levels were higher in the ABC (1300 ng/ml) than in the TEC (480 ng/ml), Wilcoxon p < 0.0001. Similarly, median cotinine levels among self-reported non-smokers were higher in the ABC (33 ng/ml) than in the TEC (5 ng/ml), Wilcoxon z = 15.63, p < 0.0001.

**Table 2:** **Summaries of urinary cotinine levels (ng/ml) by self-reported smoking status, sex, cohort and, for the ABC only, residential area**

	Self-reported non-smokers			Self-reported smokers		
	N	Median (IQR)	Range	N	Median (IQR)	Range
**ABC**						
All	124	33 (5,975)	5,3780	267	1300 (825,1810)	5,4990
Men	56	19 (9,1120)	5,2620	130	1375 (870,1910)	5,4990
Women	68	54 (10,920)	5,3780	137	1260 (725,1740)	5,4920
Remote	95	150 (12,1250)	5,3780	216	1330 (868,1785)	5,4990
Non-remote	29	7 (5,27)	5,1760	51	1200 (650,1920)	5,3180
**TEC**						
All	132	5 (5,5)	5,530	24	480 (17,928)	5,1850
Men	60	5 (5,5)	5,82	9	950 (760,1350)	14,1560
Women	72	5 (5,5)	5,530	15	72 (10,545)	5,1850

IQR: Inter-quartile Range.

Of the 124 participants in the ABC that self-reported non-smoking, 58 (47%) recorded cotinine levels higher than 50 ng/ml (the cut-off usually used for non-smokers), while of the 267 self-reported smokers, 17 (6%) had cotinine levels less than 50 ng/ml (Table 3). In contrast, 129 (98%) of the TEC self-reported non-smokers had cotinine levels of less than 50 ng/ml, with the remaining three all under 170 ng/ml. However, in self-reported smokers (n = 24), 14 had cotinine levels less than 50 ng/ml, with three of those having the minimum level (5 ng/ml) recorded. The percentage of self-reported non-smokers above the 50 ng/ml cut-off was higher in women than men in the ABC, but very similar in the TEC. The proportion of self-reported non-smokers in the ABC remote group with cotinine levels above the 50 ng/ml cut-off was over three times that seen in the ABC non-remote group.

**Table 3:** **Numbers (%) in each urinary cotinine group by self-reported smoking status and cohort, sex and, for the ABC only, residential area**

		Urinary cotinine level (ng/ml)				
		0-50	51-170	171-550	551-2100	2101+
**ABC**						
All	Smokers	17 (6)	8 (3)	22 (8)	174 (65)	46 (17)
All	Non-Smokers	66 (53)	9 (7)	6 (5)	36 (29)	7 (6)
Men	Smokers	6 (5)	3 (2)	10 (8)	84 (64)	27 (21)
Men	Non-Smokers	34 (61)	2 (4)	2 (4)	16 (29)	2 (4)
Women	Smokers	11 (8)	5 (4)	12 (9)	90 (66)	19 (14)
Women	Non-Smokers	32 (47)	7 (10)	4 (6)	20 (29)	5 (7)
Remote	Smokers	11 (5)	8 (4)	17 (8)	145 (67)	35 (16)
Remote	Non-Smokers	42 (44)	6 (6)	6 (6)	34 (36)	7 (7)
Non-remote	Smokers	6 (12)	0 (0)	5 (10)	29 (57)	11 (22)
Non-remote	Non-Smokers	24 (83)	3 (10)	0 (0)	2 (7)	0 (0)
**TEC**						
All	Smokers	8 (33)	2 (8)	4 (17)	10 (42)	0 (0)
All	Non-Smokers	129 (98)	2 (2)	1 (1)	0 (0)	0 (0)
Men	Smokers	2 (22)	0 (0)	(0)	7 (78)	0 (0)
Men	Non-Smokers	58 (97)	2 (3)	0 (0)	0 (0)	0 (0)
Women	Smokers	6 (40)	2 (13)	4 (27)	3 (20)	0 (0)
Women	Non-Smokers	71 (99)	0 (0)	1 (1)	0 (0)	0 (0)

Percentages do not add up to 100 in all cases due to rounding.

## Discussion

In this study of adolescents from Australia’s Northern Territory, we have shown striking differences in prevalence of self-reported smoking between the Indigenous and non- Indigenous cohorts. Among self-reported smokers, the median urinary cotinine measures were far greater in the ABC participants than their smoking counterparts in the TEC. Of those self-reporting that they did not smoke, nearly half of the ABC cohort had cotinine levels that suggest considerable exposure to cigarette smoke, while the percentage in the TEC with this was negligible.

Self-reported smoking prevalence was 68% in the ABC and only 14% in the TEC. The difference in prevalence rates does not appear to be due to residential location, with similar rates seen in the ABC for both the remote and non-remote populations. Further, there was little difference in mean age between the cohorts, ruling age at assessment out as a confounder. Our results are consistent with a previous study of a sample of Indigenous Australians in Arnhem Land, NT, which reported that 75% of a sample of 400 individuals aged over 16 years self-reported smoking [12]. These exceptional smoking rates in the Indigenous population of the ‘Top End’ of the NT appear to have changed little over the past twenty years, with rates consistently over 60% [26].

The use of self-reported smoking surveys in epidemiological studies is a crucial and common method used to obtain information on a major risk factor for many diseases, in addition to assessing smoking prevalence. However, it is useful to corroborate self-reported smoking data using one of a number of biochemical markers [10]. The actual choice of biochemical marker to be used will often depend on factors other than the scientific validity of the marker, but more on the feasibility of obtaining the biological material and how crucial the accurate determination of smoking status is. Measures based on tests of blood are invasive and are unable to provide an immediate assessment. Less invasive are urine tests, which for urinary cotinine can provide an almost immediate measure of smoking status. As a result, urinary cotinine is a widely used proxy metabolite for nicotine, used to distinguish between active and passive or non-smokers [27].

We used a discriminative cut-off of 50 ng/ml, in line with previous studies [14, 17, 19]. Among self-reported smokers, the average urinary cotinine measures were far greater in the ABC participants than in the TEC. Not only was there a greater prevalence of smoking in the ABC, but these results suggest heavier smoking, as well as a greater exposure to passive smoking. A previous study of the Aboriginal and Torres Strait Islander people showed that while rates of heavy smoking had decreased between 1994 and 2008 in that population, the rates of light smoking had increased [7]. Regardless, for the ABC population, the levels of smoking remain relatively high.

Of those self-reporting that they did not smoke, nearly half of the ABC cohort had cotinine levels that suggest considerable exposure to cigarette smoke, perhaps through inaccurate responses to the questionnaire, or due to high levels of passive smoking from the cigarette smoking of family and others, consistent with the overall high prevalence of smoking in this population and setting. This was in contrast to the TEC where only two percent of ‘non-smokers’ fell into this category. Gilligan et al. [18] used a cut-point of 250 ng/ml to allow for the high levels of passive smoking in Indigenous households. Even using this much higher cut-point would still have resulted in 47 (36% of the ABC self-reported non-smokers) being above the cut-point, with just one self-reported non-smoking TEC member above 250 ng/ml. A previous study of the validity of self-reported smoking in a remote Aboriginal community also used cotinine, but found good agreement between the self-report and cotinine measures [16]. n contrast to that study, the study members included in the investigation of validity in the ABC were all young adults, and it is possible that accuracy of responses to questionnaires may vary with age group. There does not appear to be a validity issue with the TEC participants. This is consistent with previous findings of different levels of validity of self-report smoking responses between ethnic groups [10]. Differences in cotinine levels between males and females may reflect different levels of smoking and passive smoking, but also including pre-menopausal groups of women with likely differential cotinine metabolism [28]. It is also possible that high levels of cotinine in light smokers may reflect compensatory smoking where individuals smoke fewer cigarettes per day, but draw back harder and absorb more nicotine per cigarette than a heavier smoker may [29].

While the differences in smoking rates between the cohorts may represent socio-economic disadvantage to some extent, it is also likely that other factors such as cultural differences in views regarding smoking initiation play an important role. This includes a long-standing historical antecedent in the way in which tobacco is currently viewed by Indigenous communities [30]. For example, it became a form of currency when obtaining payment for labour and for settling disputes [30]. In a qualitative study of Australian Aboriginal women, young girls were found to start and continue smoking as a way of attaining status and asserting their Aboriginal identity such that it is seen as belonging to the group, rather than rebelling against it [31]. A more recent study has also shown family and peer influences play a central role in smoking uptake with Indigenous youth [32], reporting high levels of smoking role models and smoking socialisation practices, consistent with the high nicotine levels in the non-smokers in our study.

Limitations of this study relate to the recruitment of the study subjects. The ABC participants were nested within a birth cohort study and are representative of the Indigenous population across the Top End of Australia in that age group. The TEC participants of a similar age were not recruited at birth and may be seen as a select group interested in their health and, perhaps, less likely to be smokers. This could introduce some selection bias, although it is unlikely to account for the large differences seen in this study. Further, while the same questionnaires were used for both populations, the ABC participants mostly needed these to be read out to them while the TEC were able to answer the questions without assistance. The greater degree of anonymity provided by self-administration of the questionnaires may have had a bearing on the truthfulness of the responses received. This could explain some of the differences in the two cohorts, but is also unlikely to completely explain the differences between the populations.

The questionnaire was limited to smoking status of participants of the ABC and TEC and did not include any information on family smoking status or environmental exposures. However, a previous study in 2008 has shown that 63% of Aboriginal and Torres Strait Islander children aged 0–14 years lived in a household with members who were daily smokers (72% in remote and 61% in non-remote areas) [33]. The NT has the worst record in overall tobacco control in Australia and was awarded the Dirty Ashtray Award for the worst performing jurisdiction in nine of the seventeen years it has been awarded since 1994 [34]. It was omitted from the national competition after it ranked last for the fourth consecutive year in 2009 [34].

## Conclusion

While tobacco control in NT has improved since 2009 [34], extra resources are needed in the NT, particularly to aid reductions in smoking prevalence in the Aboriginal population. Given the large discrepancies in smoking prevalence between the populations and persistent high levels of smoking among Indigenous young adults, this suggests that current tobacco control approaches and are in need of revision and expansion to tailor to the needs of the Indigenous population. It is likely that a broader approach encompassing social, cultural and language factors with increased attention to smoking socialisation factors is required. The cultural and language knowledge of Indigenous health workers may well be the key to these interventions [26, 35], although improvements in the reach of and access to services, and in the services themselves is also required.

## Appendix

**The questions on smoking used in the study.**

- Do you smoke tobacco?
- □ no □ used to □ yes
- If yes
- How often do you smoke?
- □ rarely □ weekly □ daily
- How many smokes would you have?
- How old were you when you started smoking?
- If used to
- How old were you when you started smoking?
- How old were you when you stopped smoking?
- Do you smoke marijuana (dope)?
- □ no □ used to □ yes
- If yes
- How often do you smoke?
- □ rarely □ weekly □ daily
- How many smokes would you have?
- How old were you when you started smoking?
- If used to
- How old were you when you started smoking?
- How old were you when you stopped smoking?
